# Supplementary material for: Study protocol for the Intraoperative Complications Assessment and Reporting with Universal Standards (ICARUS) global cross-specialty surveys and consensus
Source: PLoS One. 2024 Apr 16;19(4):e0297799. doi: 10.1371/journal.pone.0297799 (PMC11020956; doi:10.1371/journal.pone.0297799)
Supplement: Attachment — Submitted filename: replies_rev.docx [file pone.0297799.s003.docx]

Yudai Ishiyama

Academic Editor PLOSone

December 26, 2023

RE: **PONE-D-23-29646 Assessment and Reporting with Universal Standards (ICARUS) Global Cross-Specialty Surveys Among Surgeons, Anesthesiologists, Nurses, Interventional Cardiologists, and Interventional Radiologists**

Dear Editor,

Thank you for your review of our manuscript. We would like to thank the reviewers for their valuable time and input on this manuscript. Revisions have been made as per the reviewer recommendations. Below are the reviewers’ comments in bold font and our responses in plain font. We look forward to your review of the revised manuscript.

Dr. Giovanni Cacciamani, MD, MSC, FEBU

Associate Professor of Research Urology

Associate Professor of Research Radiology

Director Artificial Intelligence Center at USC Urology

USC Institute of Urology Catherine and Joseph Aresty Department of Urology

Keck School of Medicine, University of Southern California, Los Angeles, CA, USA.

Email: [Giovanni.cacciamani@med.usc.edu](mailto:Giovanni.cacciamani@med.usc.edu)

Phone: +1 (626) 4911531

**Journal Requirements:**

**When submitting your revision, we need you to address these additional requirements.**

**1. Please ensure that your manuscript meets PLOS ONE's style requirements, including those for file naming. The PLOS ONE style templates can be found at 
https://journals.plos.org/plosone/s/file?id=wjVg/PLOSOne_formatting_sample_main_body.pdf and 
https://journals.plos.org/plosone/s/file?id=ba62/PLOSOne_formatting_sample_title_authors_affiliations.pdf**

Done!

**2. Thank you for stating the following in the Competing Interests section: 
"Inderbir S. Gill has equity interest in OneLine Health and Karkinos. 
Mihir M. Desai is a consultant for Procept Biorobotics and Auris Surgical.
Andre Luis Abreu is a consultant for Koelis and Quibim, and speaker for EDAP. 
Other authors do not have any competing interests."**

**Please confirm that this does not alter your adherence to all PLOS ONE policies on sharing data and materials, by including the following statement: "This does not alter our adherence to  PLOS ONE policies on sharing data and materials.” (as detailed online in our guide for authors http://journals.plos.org/plosone/s/competing-interests).  If there are restrictions on sharing of data and/or materials, please state these. Please note that we cannot proceed with consideration of your article until this information has been declared.**

**Please include your updated Competing Interests statement in your cover letter; we will change the online submission form on your behalf.**

Done!

**3. Please note that in order to use the direct billing option the corresponding author must be affiliated with the chosen institute. Please either amend your manuscript to change the affiliation or corresponding author, or email us at plosone@plos.org with a request to remove this option.**

Yes, I confirm that the corresponding author is affiliated with the chosen institution.

**4. One of the noted authors is a group or consortium [ICARUS Global Surgical Collaboration]. In addition to naming the author group, please list the individual authors and affiliations within this group in the acknowledgments section of your manuscript. Please also indicate clearly a lead author for this group along with a contact email address.**

To reduce confusion we have deleted “ On behalf of ICARUS Global Surgical Collaboration”

**5. Please upload a copy of Figure 1, to which you refer in your text on page 1. If the figure is no longer to be included as part of the submission please remove all reference to it within the text.**

No figures are reported in the present study

**6. Please include your tables as part of your main manuscript and remove the individual files. Please note that supplementary tables (should remain/ be uploaded) as separate "supporting information" files.**

Done!

7. **Please include captions for your Supporting Information files at the end of your manuscript, and update any in-text citations to match accordingly. Please see our Supporting Information guidelines for more information: http://journals.plos.org/plosone/s/supporting-information.**

Done!

**8. Please review your reference list to ensure that it is complete and correct. If you have cited papers that have been retracted, please include the rationale for doing so in the manuscript text, or remove these references and replace them with relevant current references. Any changes to the reference list should be mentioned in the rebuttal letter that accompanies your revised manuscript. If you need to cite a retracted article, indicate the article’s retracted status in the References list and also include a citation and full reference for the retraction notice.**

Done!

**Additional Editor Comments:

This study protocol focuses on unmet clinical and research needs. Reporting intraoperative events is a complicated issue as it may directly conflict with surgeons’ benefits, as the authors mentioned.
The study protocol is neatly designed. If performed accordingly, it will contribute to improving surgical outcome research.
Please refer to the reviewers’ comments for minor issues.**

***Reply:*** Thank you for the feedback. We agree that the ICARUS initiative is essential for assessing the actual effects of intraoperative adverse events in surgical and anesthetic practices. By integrating diverse viewpoints, the ICARUS system aims to enhance our comprehension of intraoperative adverse events (IAEs) and develop methods to mitigate their influence on both patients and the wellbeing of surgeons.

----

**Reviewer #1:**

**The paper topic is of utmost interest. This study tries to cover a lack about intraoperative adverse events report, since they have a significant impact on patients and surgeons. Despite their importance, the true scale of intraoperative adverse events remains underestimated due to inadequate methods for their assessment, collection and grading. Various grading systems have been introduced , but their adoption has been limited, with important inconsistencies in reporting. The aim of this study is to present a new method of reporting intraoperative adverse events. This study has been conducted with great scientific accuracy and the paper is well written with good English form and it's ready for its final acceptance and publication.**

***Reply:*** We thank the reviewer for the comment. We believe that the ICARUS initiative is greatly needed to evaluate the real impact of intraoperative adverse events in surgery and anesthesiology. Bringing together different perspectives, the ICARUS system will help in understanding the true nature of IAEs and implementing strategies for reducing their impact on patients and surgeons' wellbeing.

-----

**Reviewer #2: Major comment

Assessment methods of intraoperative complications have not been established. This study protocol could be very helpful in improving quality of daily practice. I hope that this study will contribute to the standardization of intraoperative complications assessment and reporting system and contribute to the safety of patients as well as medical staffs.**

***Reply***: We thank the reviewer for the nice comment to our protocol.

**Specific comments

Title
#1 The title is a little long. Summarize the end part “Among Surgeons, Anesthesiologists, Nurses, Interventional Cardiologists, and Interventional Radiologists”.**

**Reply:** *We agree, and we have now shortened the title as suggested in “Study Protocol for the Intraoperative Complications Assessment and Reporting with Universal Standards (ICARUS) Global Cross-Specialty Surveys and Consensus”*

**Abstract

\# It is largely redundant with the INTRODUCTION.**

***Reply***: the abstract is reports as per PLOS author guidelines and it report carefully the methodology used. We have edited it for better explain the rational of each survey as follow:

*” Annually, about 300 million surgeries lead to significant intraoperative adverse events (iAEs), impacting patients and surgeons. Their full extent is underestimated due to flawed assessment and reporting methods. Inconsistent adoption of new grading systems and a lack of standardization, along with litigation concerns, contribute to underreporting. Only half of relevant journals provide guidelines on reporting these events, with a lack of standards in surgical literature. To address these issues, the Intraoperative Complications Assessment and Reporting with Universal Standard (ICARUS) Global Surgical Collaboration was established in 2022. The initiative involves conducting global surveys and a Delphi consensus to understand the barriers for poor reporting of iAEs, validate shared criteria for reporting, define iAEs according to surgical procedures, evaluate the existing grading systems' reliability, and identify strategies for enhancing the collection, reporting, and management of iAEs. Invitation to participate are extended to all the surgical specialties, interventional cardiology, interventional radiology, OR Staffs and anesthesiology. This effort represents an essential step towards improved patient safety and the well-being of healthcare professionals in the surgical field.”*

Please note that the abstract complies with the PLOS author guidelines.

**# With reference to previous reports, the number of surgeries performed each year is about 300 million. Please check it again.**
**Lancet 2015;385(Suppl 2):S11. doi:10.1016/S0140-6736(15)60806-6
BMJ 2020; 370 doi: https://doi.org/10.1136/bmj.m2917**

***Reply:*** We thank the reviewer for the comment, and we have edited accordingly.

**# Delete “A sample size of 2,398 respondents was calculated for the study, with invitations extended to 86,574 healthcare providers.”. This was redundant with Materials and Methods.**

**Reply:** deleted as suggested.

**Introduction

\# It is largely redundant with the ABSTRACT.**

Reply. See above. We have now edited the abstract accordingly. This is a protocol study and therefore the abstract incapsulate the rationale and the methodology used.

**# Add more specific explanation about ICARUS. In particular, mention the evaluation method.**

Reply: The evaluation method is reported properly in the method, where for each survey the authors have summarized the aim, methods and analysis to be used. We added more info regarding the ICARUS Global Surgical Collaboration project as follows” The ICAURS project is an global cross specialty initiative aiming at enhancing surgical research and practice by establishing and disseminating best-practice guidelines for assessing, collecting, grading and reporting iAEs across all surgical procedures. Its focus is not only on creating these guidelines but also on ensuring their practical applicability and effectiveness in clinical and academic settings. By standardizing iAE reporting, the project seeks to improve patient safety and outcomes in surgery, and it includes an evaluation component to assess the impact and refine the guidelines as needed”.

**MATERIALS AND METHODS

Selection and recruitment

\# On the basis of confidence level, margin of error, and the number of surgeons from WHO’s data, sample size was determined; however, you included not only surgeons but also anesthesiologist, nurses, interventional cardiologists, and IRists in data sample. Is it reasonable.**

Reply: We thank the reviewer for the comment and to judge our methodology reasonable.

**Survey 1-3

\# In these part, snowball sampling method was employed. Participants must meet the criterion on English, medical terminology in English, and current or past experience in procedure or surgeries. How was it proven that these criteria were met in this protocol? Clarify the specific criteria. Are they based only on self-reports?**

**Reply:** These replies to logic questions aim to identify the profession (as indicated in tables) and language, which are self-reported. This represents a limitation that will be discussed in detail in each paper arising from the analysis of the survey results.

**DISCUSSION

\# Please list some specific classifications previously reported, such as CLASSIC and ClassIntra. Additionally, mention any problems on them.**

**Reply.** We appreciate the reviewer's comment. Issues concerning each classification have been thoroughly discussed in a recent publication by our team (Sayegh, Aref S et al. “Severity Grading Systems for Intraoperative Adverse Events. A Systematic Review of the Literature and Citation Analysis.” *Annals of surgery* vol. 278,5 (2023): e973-e980, Reference 8 in the present study). A more in-depth discussion within the context of the present protocol for providing a reporting system (rather than a classification) would be beyond the scope of this publication and, therefore, will not be further addressed here.
